# Cutaneous infection with *Mycobacterium chelonae* in a patient with multiple sclerosis

**DOI:** 10.1016/j.idcr.2024.e02077

**Published:** 2024-09-08

**Authors:** Morteza Masoumi, Fatemeh Sakhaee, Morteza Ghazanfari Jajin, Seyed Davar Siadat, Abolfazl Fateh

**Affiliations:** a Department of Mycobacteriology and Pulmonary Research, Pasteur Institute of Iran, Tehran, Iran; b Microbiology Research Center (MRC), Pasteur Institute of Iran, Tehran, Iran

**Keywords:** Mycobacterium chelonae, Multiple sclerosis, Iran

## Abstract

Cutaneous infections caused by Mycobacterium chelonae can present with a variety of clinical symptoms, depending on the patient's immune status. Here, we report a case involving a 46-year-old woman with multiple sclerosis who developed a cutaneous infection caused by M. chelonae. The initial presentation included skin discoloration on her right wrist, which progressed to a granuloma. Following surgical intervention, the infection led to tissue atrophy and the formation of a deep cavity at the site. Upon identification of the causative pathogen, a treatment regimen consisting of clarithromycin and moxifloxacin was initiated and continued for seven months. The patient showed signs of recovery, with the swelling and deep cavity resolving; however, some redness at the site persists. The patient remains under treatment.

## Introduction

Mycobacterium chelonae is a member of the rapid-growing mycobacteria (RGM), a subset of the more common nontuberculous mycobacteria (NTM) that infrequently cause infections of the skin and soft tissues. While these infections are more commonly seen in immunocompromised individuals, they can also occur in immunocompetent patients [1], [2]. The incidence of *M. chelonae* infections is rising globally, driven by several factors, including the increasing number of surgical and cosmetic procedures, the expanded use of immunosuppressive drugs, and advancements in techniques for isolating and identifying these microorganisms [3]. Despite the increasing number of cases, misdiagnoses and delayed detection remain common. Consequently, it is essential to recognize the atypical clinical presentations of cutaneous NTM infections to ensure timely and accurate diagnosis [4]. In this study, we reported a case of a right wrist injury caused by *M. chelonae* in a patient with multiple sclerosis (MS).

## Case presentation

The patient is a 46-year-old woman from Canada who has been living with multiple sclerosis (MS) since her diagnosis in 2003. Initially, she was treated with interferon beta-1a. However, nearly seven years ago, her neurologist switched her medication to teriflunomide (14 mg/day) due to worsening blurred vision. Following the change in treatment, her MS attacks subsided, but the blurred vision persisted without further deterioration.

**Fig. 1:**
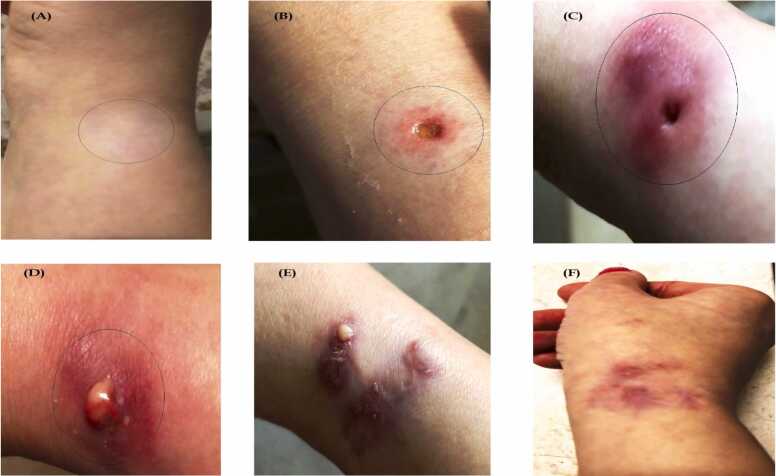
Stages of disease progression to treatment. **(A)** Discoloration of the skin on the right wrist (appearing white); **(B)** Initial biopsy showing red, swollen, painful, and purulent infection; **(C)** Tissue atrophy and formation of a deep cavity post-surgery; **(D)** Vesiculation and wound infection following surgery; (E) Wound drying after antibiotic treatment; **(F)** Wound healing with residual redness.

In September 2022, the patient consulted her physician due to discoloration of the skin on her right wrist. After a thorough examination, she was prescribed several topical treatments, including zinc-based creams, but none proved effective. In February 2023, a dermatologist recommended a biopsy to definitively diagnose the infection. Following the biopsy, the lack of sutures and the open wound at the biopsy site led to the development of redness, swelling, pain, and purulent infection within four days. During the biopsy, the doctor did not use gloves, and the punch used was not sterile. This lack of proper infection control may have contributed to the contamination. Following the biopsy, the patient was prescribed several medications, including Co-amoxiclav (625 mg) and clindamycin (300 mg), along with topical ointments such as ARAZLO® (tazarotene lotion 0.045 %) and mupirocin ointment. However, these treatments proved ineffective, and the lesion continued to spread. Subsequently, granulomata developed throughout the dermis at the lesion site, accompanied by fibrosis. In April 2023, the granulomata were surgically excised, and the removed mass was sent to the laboratory for further examination to investigate the possibility of an infection. Following the surgery and removal of the granuloma, the tissue atrophied, and a deep cavity developed. Despite treatment with cloxacillin 500 mg and cephalexin 500 mg administered three times daily, the infection continued to spread (Fig. 1A-F).

After seven months, she traveled to Iran and visited Razi Skin Hospital in Tehran. Her physician ordered a range of tests, including a Leishmaniasis test, smear, culture, and polymerase chain reaction (PCR) for mycobacterial diagnosis. Additionally, the physician requested tests for deep mycosis, chest radiography, and a comprehensive blood panel, which included white blood cell count, C-reactive protein, erythrocyte sedimentation rate, platelet count, and liver enzyme levels.

The tissue sample from the wrist was sent to the Pasteur Institute of Iran for evaluation of a mycobacterial infection. The smear test for acid-fast bacilli (AFB) yielded a negative result. Additionally, the results of her clinical tests were within normal ranges. The histopathological examination revealed significant cutaneous inflammation characterized by an abundance of neutrophils, with scattered lymphocytes and histiocytes. Ziehl-Neelsen staining confirmed the presence of acid-fast bacilli (AFB) in the tissue.

The biopsy sample was cultured on Lowenstein-Jensen medium. After four days, the culture yielded smooth, clear to cream-colored colonies indicative of RGM. Biochemical testing results showed positive reactions for arylsulfatase, thermostable catalase, urea hydrolysis, and tellurite reduction, while being negative for niacin accumulation, nitrate reduction, iron uptake, growth in the presence of 5 % NaCl, and on MacConkey agar without crystal violet. Molecular detection was performed using PCR targeting three genes: *hsp65*, *rpoB*, and *16S rDNA*, as previously described [5]. The results of both biochemical and molecular tests confirmed that the isolate was *M. chelonae*.

Following the criteria established by the Clinical and Laboratory Standards Institute (CLSI), a drug susceptibility test (DST) was conducted. *M. chelonae* was found to be susceptible to tobramycin, linezolid, clarithromycin, moxifloxacin, and imipenem. Based on the in vitro susceptibility test results, the patient was treated with clarithromycin 500 mg twice daily and moxifloxacin 400 mg once daily for a duration of 7 months. The patient is still undergoing treatment, and there has been significant improvement. However, some swelling and redness is observed in the right wrist.

## Discussion

To the best of our knowledge, this is the first documented case of a skin infection caused by *M. chelonae* in a patient with MS. Current MS treatments, including immunomodulatory drugs and immunosuppressants, influence the immune system in various ways. Teriflunomide, for example, is known to suppress immune system activity to varying extents. Teriflunomide effectively inhibits B lymphocyte proliferation by directly targeting dihydroorotate dehydrogenase and reducing lipopolysaccharide-induced proliferation, which is mediated through the release of immunoglobulin M (IgM) by B cells. Furthermore, the class switch of immunoglobulin to IgG1, driven by interleukin 4, inhibits B-cell proliferation independently of teriflunomide’s action. In other words, teriflunomide alters the interaction between T and B cells, leading B cells to produce IgG antibodies instead of IgM antibodies, representing a class switch in antibody production. Teriflunomide effectively disrupts the interaction between B and T cells, thereby inhibiting T lymphocyte-dependent antibody production. Additionally, teriflunomide has been associated with alterations in T lymphocyte function-associated antigen 1 and CD43, as well as disruptions in T-lymphocyte activation in response to antigen-presenting cells. This leads to impaired T cell activation due to the disruption of calcium signaling, which is essential for effective intercellular communication. Given this, medications used to modify MS may increase the risk of opportunistic infections, such as mycobacterial infections, or potentially reactivate existing infections due to their impact on cellular immunity [6], [7], [8].

*M. chelonae* is an opportunistic pathogen known to cause both localized and disseminated skin infections, particularly in individuals with compromised immune systems. In this case, the patient's history of teriflunomide therapy, which can suppress immune function, is a crucial factor to consider. This medication may have contributed to the development of the infection by weakening the patient’s immune response, thereby allowing the pathogen to establish and proliferate [4].

Several studies have demonstrated that certain MS medications can increase the risk of infections, including tuberculosis, due to their effects on the immune system. These drugs can potentially lead to both latent tuberculosis infection and active disease progression. Consequently, it is important for clinical practice to include routine screening for latent tuberculosis in MS patients and to evaluate risk factors that might lead to the progression of latent infection to active tuberculosis. This proactive approach can help in early identification and management of potential infections associated with MS treatments [7].

The clinical presentation of NTM cutaneous infections is typically nonspecific and varies widely. Infections caused by *M. chelonae* often present as disseminated skin lesions. However, they can also appear as localized cellulitis, abscesses, ulcers, subcutaneous nodules, or, in rare instances, deep cavities, as observed in our patient [2].

The patient was treated with clarithromycin and moxifloxacin. Treatment for NTM infections differs from that for Mycobacterium tuberculosis due to the inherent resistance of NTMs to standard antituberculous medications and metronidazole. Resistance to ciprofloxacin, cefotaxime, sulfamethoxazole, and doxycycline has been documented. Although there are no randomized controlled trials establishing a specific treatment regimen for NTM infections, clarithromycin is commonly used with limited reports of resistance. To mitigate resistance, it is advisable to use clarithromycin in combination with another antibiotic, such as moxifloxacin or levofloxacin. The recommended duration of therapy generally ranges from four to six months, or alternatively, from the time of diagnosis until four to six weeks after the lesion has healed [9], [10].

## CRediT authorship contribution statement

**Morteza Masoumi:** Data curation, Conceptualization. **Fatemeh Sakhaee:** Methodology, Investigation. **Morteza Ghazanfari Jajin:** Software. **Seyed Davar Siadat:** Writing – review & editing. **Abolfazl Fateh:** Writing – review & editing, Writing – original draft, Supervision, Software.

## Ethics approval

This study was approved by the Ethics Committee of Pasteur Institute of Iran (IR.PII.REC.1402.027).

## Funding sources

No funding was received.

## Informed consent

Patients’ consents were obtained.

## Declaration of Competing Interest

The authors declare that they have no known competing financial interests or personal relationships that could have appeared to influence the work reported in this paper.
